# Association of *PCSK1* and *PPARG1* Allelic Variants with Obesity and Metabolic Syndrome in Mexican Adults

**DOI:** 10.3390/genes14091775

**Published:** 2023-09-08

**Authors:** Jorge Velazquez-Roman, Uriel A. Angulo-Zamudio, Nidia Leon-Sicairos, Hector Flores-Villaseñor, Miriam Benitez-Baez, Ana Espinoza-Salomón, Alejandra Karam-León, Hugo Villamil-Ramírez, Samuel Canizales-Quinteros, Luis Macías-Kauffer, Jose Monroy-Higuera, Erika Acosta-Smith, Adrian Canizalez-Roman

**Affiliations:** 1 School of Medicine, Autonomous University of Sinaloa, Culiacan Sinaloa 80019, Mexico; jorgevelazquezroman@uas.edu.mx (J.V.-R.); uriel.zamudio@uas.edu.mx (U.A.A.-Z.); nidialeon@uas.edu.mx (N.L.-S.); floreshector@uas.edu.mx (H.F.-V.); anasaileespinoza@gmail.com (A.E.-S.); erika.acosta@uas.edu.mx (E.A.-S.); 2 Pediatric Hospital of Sinaloa, Constitución 530, Jorge Almada, Culiacan Sinaloa 80200, Mexico; 3 The Sinaloa State Public Health Laboratory, Secretariat of Health, Culiacan Sinaloa 80020, Mexico; 4 Programa de Doctorado, Posgrado Integral en Biotecnología, FCQB, UAS, Culiacan Sinaloa 80013, Mexico; miriam_150@msn.com (M.B.-B.); alejandrakaram@hotmail.com (A.K.-L.); monroyantonio121@gmail.com (J.M.-H.); 5 Unidad de Genómica de Poblaciones Aplicada a la Salud, Facultad de Química, UNAM/INMEGEN, Mexico City 04510, Mexico; hugo_villamil@hotmail.com (H.V.-R.); cani@unam.mx (S.C.-Q.); luisrmacias@gmail.com (L.M.-K.); 6 The Women’s Hospital, Secretariat of Health, Culiacan Sinaloa 80020, Mexico

**Keywords:** SNP, *PCSK1*, *PPARG1*, obesity, metabolic syndrome

## Abstract

Metabolic diseases, including obesity, diabetes, and metabolic syndrome, are among the most important public health challenges worldwide. Metabolic diseases are classified as multifactorial diseases in which genetic variants such as single-nucleotide polymorphisms (SNPs) may play an important role. The present study aimed to identify associations linking allelic variants of the *PCSK1*, *TMEM18*, *GPX5*, *ZPR1*, *ZBTB16*, and *PPARG1* genes with anthropometric and biochemical traits and metabolic diseases (obesity or metabolic syndrome) in an adult population from northwestern Mexico. Methods: Blood samples were collected from 523 subjects, including 247 with normal weight, 276 with obesity, and 147 with metabolic syndrome. Anthropometric and biochemical characteristics were recorded, and single-nucleotide polymorphisms (SNPs) were genotyped by real-time PCR. Results: *PCSK1* was significantly (*p* < 0.05) associated with BMI, weight, and waist-to-hip ratio; TMEM18 was significantly associated with systolic blood pressure and triglyceride levels; *GPX5* was significantly associated with HDL cholesterol levels. In addition, *PCSK1* was associated with obesity (*p* = 1.0 × 10^−4^) and metabolic syndrome (*p* = 3.0 × 10^−3^), whereas *PPARG1* was associated with obesity (*p* = 0.044). Conclusions: The associations found in this study, mainly between allelic variants of *PCSK1* and metabolic traits, obesity, and metabolic syndrome, may represent a risk for developing metabolic diseases in adult subjects from northwestern Mexico.

## 1. Introduction

Today, metabolic diseases are one of the most important health problems worldwide, with obesity, diabetes, and metabolic syndrome affecting most of the population. Obesity is classified as an abnormal accumulation of fat that causes organic changes. Approximately 10–15% of the world’s population suffers from obesity; in Mexico, 75.2% of the population is obese or overweight, and the state of Sinaloa is among the 10 Mexican states with the highest prevalence of this disease [1,2]. Metabolic syndrome is a clinical condition that includes central and abdominal obesity, systemic hypertension, insulin resistance, and atherogenic dyslipidemia, and it is estimated that 34% of people with obesity have metabolic syndrome [3]. Metabolic diseases are classified as multifactorial diseases in which genetic variants such as single-nucleotide polymorphisms (SNPs) may significantly contribute to the development of these diseases.

The study of the genetics of metabolic diseases began with genome-wide association studies (GWASs), which found a large number of SNPs (more than 120 genes) associated with obesity or metabolic syndrome, mainly in European populations [4]. For example, the proprotein convertase subtilisin/kexin type 1 (PCSK1) gene is responsible for the activation of several neuropeptides and hormones involved in thermogenesis and feeding. In fact, the PCSK1 SNP is the third most important gene associated with obesity; this gene is one of the first genes recognized as a monogenic cause of obesity [5,6,7]. The zinc finger BTB domain-containing 16 (ZBTB16) gene is involved in the thermogenic process, as well as fatty acid oxidation in brown adipocytes, and genetic variants of ZBTB16 have been associated with changes in anthropometric parameters and lipid levels in the population. They are a risk factor for developing metabolic diseases [8,9]. ZPR1 is a zinc finger protein that acts as a transcription factor associated with peroxisome proliferator-activated receptor γ; this protein also interacts with apolipoprotein A5, which helps regulate postprandial triglycerides. SNPs in this gene are associated with elevated triglyceride and glucose levels and altered insulin sensitivity, and they have been implicated in several metabolic disorders (e.g., metabolic syndrome and type 2 diabetes mellitus) [10,11,12]. The peroxisome proliferator-activated receptor γ (*PPARG*) gene is a transcription factor that regulates genes involved in metabolic homeostasis and energy expenditure and storage; in addition, *PPARG* is the master regulator of adipocyte differentiation, and it modulates metabolism and inflammation in immune cells [13]. Its polymorphism has been associated with altered energy expenditure, leading to increased body mass index (BMI) and obesity [14]. The transmembrane protein 18 (TMEM18) gene is a nuclear membrane-localized protein in various brain regions, including the hypothalamus, that controls feeding behavior, and its function is related to the transcriptional repressor that binds to a specific ssDNA sequence. SNPs in this gene are associated with changes in satiety and obesity, but the mechanisms remain unclear [15,16].

Studies of allelic variants of *PCSK1* rs6235 G allele, *TMEM18* rs6548238 C allele, *GPX5* rs445870 G allele, *ZPR1* rs964184 G allele, *ZBTB16* rs7106340 T allele, and *PPARG1* rs3856806 T allele are limited or absent in adults from northwestern Mexico, and their association with metabolic diseases is not well known. Furthermore, the possible associations found in this study could help to detect genetic risk factors for the development of metabolic diseases (obesity and/or metabolic syndrome) in our population; moreover, we could lay the groundwork for new specific treatments against metabolic diseases for our population taking into account genetic profiles. Therefore, this work aimed to identify individual associations of six previously described SNPs with anthropometric and biochemical traits and with metabolic diseases such as obesity or metabolic syndrome in a population from northwestern Mexico.

## 2. Materials and Methods

### 2.1. Study Subjects

The methodology of Velazquez-Roman et al. was followed with some modifications [17]. A nested case–control analysis included subjects with normal weight and obesity or metabolic syndrome. According to the frequency of *FTO* rs9939609 in Mexico, the sample size was calculated, taking an allele frequency of 0.23 in an additive model; a minimum of 250 participants per group was needed.

Inclusion criteria: Subjects had to be older than 30 years and residents of the state of Sinaloa. As for the subjects with obesity and normal weight, they were those who met the classification standard according to the World Health Organization (WHO) criteria (normal weight BMI < 25 kg/m^2^; obesity BMI > 30 kg/m^2^), included in their respective study group [1]. On the other hand, to classify subjects with metabolic syndrome, the Adult Treatment Panel III (ATP III) and the American Heart Association/National Heart, Lung, and Blood Institute Scientific Statement criteria were used, in which, if a patient presents three of the following characteristics, they were classified as subject with metabolic syndrome: waist circumference, female > 88 cm, male > 102 cm; blood pressure, systolic < 130 mmHg, diastolic < 85 mmHg; HDL cholesterol, female < 40 mg/dL, male < 50 mg/dL; triglycerides > 150 mg/dL; glucose > 100 mg/dL [18,19]. Subjects with type 2 diabetes or any endocrinologic disease, such as those using steroid medications, hypoglycemic agents, and/or drugs of abuse, were excluded.

### 2.2. Measurement of Biochemical and Anthropometric Traits

Firstly, blood samples were taken from all participants. To take blood samples, patients signed an informed consent form; additionally, this project was approved by the Women’s Hospital of Sinaloa ethics committee, Health Minister No. 202029-07. From blood samples, biochemical traits were measured: glucose, total cholesterol, low-density lipoprotein cholesterol (LDL-C), high-density lipoprotein cholesterol (HDL-C), triglycerides, and creatinine. On the other hand, anthropometric traits were measured: height, weight, and waist and hip circumferences [20]. Moreover, BMI and waist-hip ratio (WHR) were calculated, and blood pressure was measured.

### 2.3. Genotyping

To analyze the study subjects’ DNA, it was isolated from peripheral leukocytes using a commercial kit (QIAmp 96 DNA Blood Kit; Qiagen, Hilden, Germany) according to the manufacturer’s instructions. Then, Nanodrop 2000 (ThermoFisher Scientific, Waltham, MA, USA) was used to determine the amount and purity of DNA extracted from the samples. Purity around 1.8 and 2.0 was considered acceptable. *PCSK1* rs6235, *TMEM18* rs6548238, *GPX5* rs445870, *ZPR1* rs964184, *ZBTB16* rs7106340, and *PPARG1* rs3856806 were genotyped using TaqMan probes (Applied Biosystems, Foster City, CA, USA) (Table S1) and a LightCycler^®^ 96 II instrument (Roche, Rotkreuz, Switzerland). For the PCR reaction, 10 ng/mL of DNA was used in a master mix (enzyme, buffer, magnesium chloride, dNTP, and nuclease-free water) (Roche, Basel, Switzerland); preincubation of 95 °C for 600 s was applied, followed by 45 cycles of DNA amplification at 60 °C for 60 s, and 95 °C for 10 s. Approximately 5% of the samples were duplicates, and genotyping was concordant in all cases.

### 2.4. Statistical Analysis

Means ± standard deviation (SD) and percentages were used to present the descriptive characteristics of the subjects. The Kolmogorov–Smirnov test was used to determine the distribution of the data. The Mann–Whitney U test was used to analyze differences between groups. Linear regression was used to determine the effect of SNPs on anthropometric and biochemical traits, adjusting for sex and age (anthropometric traits) or sex, age, and BMI (biochemical traits). In contrast, logistic regression was used to determine associations between SNPs and metabolic diseases, adjusting for age, sex, and site for obesity and metabolic syndrome analyses. All analyses were under an additive model. The odds ratio (OR) and 95% confidence index (95% CI) were calculated; a *p*-value ≤ 0.05 was taken as statistically significant. SPSS (version 20.0; Chicago, IL, USA) was used for statistical analyses. No deviation from the Hardy–Weinberg equilibrium was observed for any SNP in any group (*p* > 0.05).

## 3. Results

### 3.1. Characteristics of the Subjects

A total of 523 subjects (247 normal weight and 276 obese) were included in the study, of whom 67.3% (352/523) were female and 32.7% (171/523) were male. The characteristics of the study population stratified by body mass index are summarized in Table 1. In addition, 147/523 subjects had metabolic syndrome (Table 2). Anthropometric and biochemical characteristics were compared between normal-weight and obese subjects, and between subjects without metabolic syndrome and subjects with metabolic syndrome. In general, most anthropometric and clinical characteristics such as weight, BMI, waist circumference, heart rate, systolic blood pressure, diastolic blood pressure, and glucose, cholesterol, and triglyceride levels were higher in subjects with obesity or metabolic syndrome than in subjects with normal weight and without metabolic syndrome (*p* < 0.05) (Table 1 and Table 2).

### 3.2. Association of Several SNPs with Biochemical and Anthropometric Characteristics of the Study Subjects

The presence of six different SNPs was explored in the adult study population of northwestern Mexico, and their frequencies in the study subject are shown in Table 3. The most frequent allelic variant was *TMEM18* (84.4%), followed by *GPX5* (40.5%), *PCSK1* (31.4%), *ZPR1* and *ZBTB16* (24.7% each), and *PPARG1* (5.7%).

Five individual SNPs previously mentioned were significantly associated with anthropometric or biochemical parameters of the whole population (Table 4 and Table 5). The G rs6235 allele of *PCSK1* was significantly associated with increased anthropometric traits, such as BMI around 1.04 kg/m^2^, weight 2.186 kg, waist circumference 2.275 cm, and WHR 0.011 (Table 4). In addition, the C allele of *TMEM18* rs6548238 was associated with anthropometric and biochemical traits. This SNP increased systolic pressure by 3218 mm/Hg and triglyceride levels by 19,377 mg/dL (Table 4 and Table 5). Another SNP influencing biochemical traits was the T rs3856806 allele of *PPARG1*, which was associated with higher total cholesterol levels, which increased to 14,310 mg/dL (Table 5). In addition, the presence of the G allele rs445870 of *GPX5* significantly decreased HDL cholesterol by 1.655 mg/dL (Table 5). The remaining SNPs (*ZBTB16* rs7106340 T allele and *ZPR1* rs964184 G allele) were not significantly associated with anthropometric or biochemical traits in the studied population.

### 3.3. Individual Associations of Several SNPs with Metabolic Diseases: Obesity and Metabolic Syndrome

After analyzing the association of the six SNPs with anthropometric and biochemical traits in the general population, the association of the SNPs with obesity and/or metabolic syndrome was investigated. Of the six SNPs studied, two showed individual associations with metabolic diseases; one of them was the G rs6235 allele of *PCSK1*, which had an allele frequency of 39.3% in obese individuals versus 29.1% in individuals of normal weight and was significantly associated with obesity, increasing 1.54 times the probability of suffering from this pathology (95% CI: 1.22–1.92; *p*: 1.0 × 10^−4^) (Table 6). In addition, the G rs6235 allele of *PCSK1* was the only allele associated with metabolic syndrome in adults from northwestern Mexico, increasing 1.50 times the probability of having this metabolic disease (95% CI: 1.15–1.97; *p*: 3.0 × 10^−3^) (Table 7). Another SNP associated with obesity in this population-based study was the T rs3856806 allele of *PPARG1*, with 1.78-fold increased odds of obesity (95% CI: 1.01–3.10; *p*: 0.044). The frequency of this allele was 9.4% in obese individuals versus 6.4% in normal-weight individuals (Table 6).

## 4. Discussion

Obesity and metabolic syndrome are multifactorial diseases in which genetic components may play an important role. In this work, we found that the allelic variants *PCSK1* rs6235 G allele, *TMEM18* rs6548238 C allele, *PPARG1* rs3856806 T allele, and *GPX5* rs445870 G allele were associated with anthropometric and biochemical changes in subjects from the state of Sinaloa. In addition, the *PCSK1* rs6235 G allele was associated with obesity and metabolic syndrome, and the *PPARG1* rs3856806 T allele was associated with obesity.

The allele frequencies of some SNPs In this work were higher compared to other ethnic groups, such as the G allele of *PCSK1* rs6235 (19.99%) and the G allele of *GPX5* rs445870 (33%) [21,22], but the frequencies of other SNPs were similar to those of different populations, such as the C allele of *TMEM18* rs6548238 [23,24]. The differences in SNP frequencies compared to our study may be because the other studies were conducted in different populations, such as Asians or Europeans.

The presence of some SNPs has been observed to be associated with changes in biochemical or anthropometric traits in some populations, and this study was no exception. The G rs6235 allele of *PCSK1* was associated with increased anthropometric traits, including BMI, weight, waist circumference, and WHR. These results are consistent with other studies; for example, in the Chinese population, an allelic variant of *PCSK1* rs6234 was associated with increased BMI and waist circumference [25,26], and, in the young European-American population, these SNPs were associated with increased BMI [27]. Similar results were found in British and Mexican subjects [28,29]. The increase in BMI, weight, waist circumference, and HR associated with the *PCSK1* SNP in this population could be because this SNP is strongly associated with obesity due to alterations in thermogenesis and feeding, among other metabolic changes, increasing the anthropometric characteristics mentioned above [26].

On the other hand, no previous study has reported an association between the C allele of *TMEM18* rs6548238 and increased systolic blood pressure in adults, as observed in this study; this increase in systolic blood pressure may be related to the ability of *TMEM18* to cause obesity, but the mechanisms are still unclear.

Regarding biochemical parameters, several studies have shown that *PPARG1* SNPs could increase cholesterol levels in different populations, and our results are consistent with these studies [30]. This association could be because *PPARG* is related to cholesterol efflux in cells, and altering this gene could increase blood cholesterol levels [31]. Triglyceride level was another trait associated with the C allele of *TMEM18* rs6548238 that was altered in this study, a phenomenon also observed in the Chinese population [32]; this effect may be related to its ability to induce obesity. As for the G allele of *GPX5* rs445870, there are no previous reports on its association with reduced HDL cholesterol in adults, and its mechanism is unknown.

Regarding metabolic diseases, in this study, we analyzed the individual associations of six SNPs with obesity and metabolic syndrome, among which the G rs6235 allele of *PCSK1* was associated with obesity. This association has also been identified in different populations, such as European subjects. A study conducted on Danish subjects analyzed 3074 obese and 2790 non-obese subjects and found associations between *PCSK1* rs6232 and rs6235 and obesity. In addition, in Swiss subjects, an association between rs6235 and rs6232 and obesity was found in a comparison of 551 obese Swiss class III individuals and 542 randomly selected blood donors. A comparison between 532 non-obese French young adults and 505 obese French children showed an association between *PCSK1* rs6232 (OR: 1.57, *p*: 0.009) and rs6235 (OR: 1.50 (1.23, *p*: 0.00003) and obesity, results consistent with this study [33]. Reported associations between *PCSK1* allelic variants (rs6232 and rs6235) in European-American (OR: 1.71, *p*: 0.018) and African-American (OR: 1.47, *p*: 0.018) individuals and obesity, but not in Hispanic individuals [27]. In addition, allelic variants of *PCSK1* (rs6232 and rs6235) were also studied in Mexican subjects; 802 non-obese and 404 obese children and 562 non-obese and 614 obese adults from Mexico City were analyzed. The rs6232 SNP was associated with obesity in children (OR: 3.78, *p*: 0.0003) and with class III obesity in adults (OR: 2.61, *p*: 0.02), consistent with our findings [21]. Furthermore, associations between *PCSK1* allelic variants and obesity have been reported in Chinese and Taiwanese subjects [25,34,35].

Mutation in the *PCSK1* gene is sufficient to cause obesity, as this gene is part of a family of genes classified as responsible for monogenic obesity; in fact, alterations in this gene are associated with early onset obesity, severe intestinal malabsorption, and hyperphagia [36]. The strong association between *PCSK1* and obesity is due to its function; mutation in this gene leads to the misprocessing of melanocortin peptides, which alters the role of hormones and neuropeptides important for thermogenesis and feeding and involved in glucose homeostasis, fat oxidation, and energy expenditure [5,6,7,37].

In addition, the T rs3856806 allele of *PPARG1* was also associated with obesity in adults from northwestern Mexico. Vaisam-Castro G et al. (2021) also found an association between *PPARG* rs1801282 and obesity in Brazilians [38]. In contrast, other studies in Portuguese women [39] and children [40] found no association between *PPARG* allelic variants and obesity. In a previous study performed by our team on adults from northwestern Mexico, we did not find an individual association with obesity, but the allelic variant analyzed was *PPARG* rs1801282 [17]. However, both *PPARG* rs1801282 and rs3856806) are in linkage disequilibrium [41]; rs1801282 is the most studied, and some work has associated this polymorphism with obesity or obesity-related traits, whereas rs3856806 has been associated with protective activity in dyslipidemia [38,42]. Further studies are needed to establish individual associations between *PPARG* allelic variants and obesity. The mechanisms of *PPARG1* that were associated with obesity may be due to changes in energy expenditure and energy storage [42].

On the other hand, the G rs6235 allele of *PCSK1* showed an individual association with this metabolic disorder. The association between the G rs6235 allele of *PCSK1* and metabolic syndrome might be confounded in this population because this allelic variant increased some anthropometric components of metabolic syndrome, such as BMI, weight, waist circumference, and WHR. Interestingly, no previous reports have associated the G rs6235 allele of *PCSK1* with metabolic syndrome, only with some components of metabolic syndrome. For example, in a study analyzing 27,786 European subjects, *PCSK1* rs6235 and rs6232 were associated with increased waist circumference and hip ratio in men and women [7]. Three *PCSK1* variants (rs10515237, rs6232, rs43636321, and rs3792747) were associated with increased systolic and diastolic blood pressure and risk of hypertension in 7869 European subjects [43]. In addition, *PCSK1* rs6232 was associated with increased fasting glucose levels [44,45]. In addition, *PCSK1* variants may affect HDL cholesterol; one study showed that the livers of *PCSK1* mutant mice had 2.0-fold lower levels of serum apolipoprotein A1, the primary component of HDL, but HDL cholesterol concentration was unaffected [46]. The effect of *PCSK1* on metabolic syndrome traits could be due to (1) its activity in energy metabolism, as mentioned above, (2) regulation of blood pressure by the renin–angiotensin–aldosterone system (RAAS), as *PCSK1* is involved in the processing of prorenin to renin, and variants in this gene could affect the RAAS [47], or (3) the involvement of *PCSK1* in glucose and lipid metabolism by modulating insulin and APOA1 production [48,49]. On the other hand, the lack of association of the other SNPs (*TMEM18* rs6548238 C allele, *GPX5* rs445870 G allele, *ZPR1* rs964184 G allele, and *ZBTB16* rs7106340 T allele) with metabolic diseases (obesity or metabolic syndrome) in this work could have been influenced by the sample size.

Within SNP research, there are two important approaches: (1) diagnosis, where the more SNPs associated with specific diseases (in this case, obesity and metabolic syndrome) there are in a population, the more genetic profiles can be developed to identify at an early stage subjects with a high probability of developing metabolic diseases; (2) treatment, where having a genetic profile of SNPs associated with a particular disease or trait, we can choose the best therapy to treat the metabolic disease; in this sense, SNP research is in the process of developing personalized therapies according to the genetic profile [50].

To our knowledge, this is the first report linking the C allele of *TMEM18* rs6548238 and the G allele of *GPX5* rs445870 with increased systolic blood pressure and decreased HDL cholesterol, respectively, in adults, but the mechanism remains to be elucidated. In addition, this is the first report of an association between the G rs6235 allele of *PCSK1* and metabolic syndrome based on international standards.

Limitations of this study included the sample size (with a larger sample, more associations are likely to be found) and the stratification of the population (the same *n* in each study group of normal weight versus obese and nonmetabolic syndrome versus metabolic syndrome might help to find more associations between SNPs and metabolic syndrome), as the data were not adjusted for admixture (taking into account the geographic origin based on the genetic ancestry of the study subjects might provide different associations). In addition, fewer subjects were analyzed for the G rs1801282 allele of *PPARG1* compared with the other SNPs because of technical problems that prevented analysis of the entire sample.

## 5. Conclusions

This study provides evidence that allelic variants were associated with changes in anthropometric and biochemical traits in adults from northwestern Mexico. In addition, the G allele of *PCSK1* SNP rs6235 was individually associated with obesity and metabolic syndrome, and the G allele of *PPARG* rs1801282 was associated with obesity. Our findings support that the G allele rs6235 of *PCSK1* may be a determinant factor in developing obesity and obesity-related metabolic trait changes in Mexican subjects. However, further studies with larger sample sizes are needed to confirm this association.

## Figures and Tables

**Table 1:** Anthropometric and biochemical characteristics of the study groups.

	Normal Weight	Subjects with Obesity	
	*n =* 247	*n =* 276	
Characteristics	Mean (SD)	Mean (SD)	*p* Value
Age (years)	39.23 ± 9.84	43.68 ± 10.75	3.1 × 10^−7^ *
Height (m)	1.65 ± 0.09	1.62 ± 0.08	1.5 × 10^−5^ *
Weight (kg)	64.05 ± 10.50	89.87 ± 17.36	1.3 × 10^−62^ *
BMI (kg/m^2^)	23.14 ± 1.92	34.02 ± 5.66	1.22 × 10^−85^ *
Waist circumference (cm)	79.24 ± 9.17	106.03 ± 15.38	2.7 × 10^−70^ *
Hip circumference (cm)	97.93 ± 7.88	119.32 ± 61.95	3.32 × 10^−62^ *
WHR	0.809 ± 0.079	0.915 ± 0.109	5.32 × 10^−33^ *
Diastolic pressure (mm/Hg)	70.61 ± 10.25	79.65 ± 11.47	1.5 × 10^−20^ *
Systolic pressure (mm/Hg)	110.61 ± 14.42	126.22 ± 30.94	4.2 × 10^−21^ *
Cholesterol (mg/dL)	188.76 ± 53.63	179.82 ± 46.86	0.062
LDL (mg/dL)	125.30 ± 43.69	117.59 ± 38.15	0.269
HDL (mg/dL)	54.27 ± 19.62	38.04 ± 14.58	1.89^−26^ *
Glucose (mg/dL)	89.82 ± 13.13	94.82 ± 14.54	0.035 *
Triglycerides (mg/dL)	105.77 ± 74.44	156.59 ± 80.03	7.1 × 10^−21^ *
Creatinine (mg/dL)	1.347 ± 9.807	0.637 ± 0.352	4.2 × 10^−9^ *

Abbreviations: m: meters; cm: centimeters; kg: kilograms; mm: millimeters; Hg: mercury; mg: milligrams; dL: deciliters, WHR: waist-to-hip ratio; SE: standard deviation. The mean and standard deviation were obtained by means of a frequency analysis. Normal values: BMI: 20–25 kg/m^2^, blood pressure: 120/80 mm/Hg, cholesterol: <200 mg/dL, LDL: <130 mg/dL, HDL: greater than 50 mg/dL in women and 40 mg/dL in men, triglycerides: <150 mg/dL, glucose: <100 mg/dL, creatinine: 0.7–1.3 mg/dL. * Statistical significance vs. lean. Significance of *p* was taken at a value < 0.05.

**Table 2:** Anthropometric and biochemical characteristics of subjects with and without metabolic syndrome.

	Nonmetabolic Syndrome	Metabolic Syndrome	
	*n* = 376	*n* = 147	
Traits	Mean (SD)	Mean (SD)	*p* Value
Age (years)	39.89 ± 10.00	45.95 ± 10.74	2.4 × 10^−9^ *
Height (m)	1.64 ± 0.093	1.62 ± 0.08	9.9 × 10^−4^ *
Weight (kg)	72.34 ± 17.92	91.93 ± 16.27	5.9 × 10^−28^ *
BMI (kg/m^2^)	26.59 ± 6.21	34.64 ± 5.28	1.1 × 10^−37^ *
Waist circumference (cm)	87.27 ± 16.33	108.90 ± 14.19	1.8 × 10^−37^ *
Hip circumference (cm)	106.28 ± 53.96	116.60 ± 12.64	1.8 × 10^−38^ *
WHR	0.838 ± 0.11	0.935 ± 0.097	1.4 × 10^−21^ *
Diastolic pressure (mm/Hg)	72.21 ± 9.96	82.90 ± 11.36	2.8 × 10^−20^ *
Systolic pressure (mm/Hg)	114.28 ± 26.70	129.94 ± 17.69	9.5 × 10^−23^ *
Cholesterol (mg/dL)	182.09 ± 50.51	188.78 ± 48.97	0.055
LDL (mg/dL)	119.34 ± 41.41	124.37 ± 38.60	0.061
HDL (mg/dL)	49.77 ± 18.60	34.41 ± 11.16	3.8 × 10^−22^ *
Glucose (mg/dL)	89.60 ± 14.43	94.82 ± 14.71	0.002 *
Triglycerides (mg/dL)	109.45 ± 66.05	193.27 ± 85.01	7.0 × 10^−33^ *
Creatinine (mg/dL)	0.680 ± 0.194	0.651 ± 0.448	2.7 × 10^−4^ *

Abbreviations: m, meters; cm, centimeters; kg, kilograms; mm, millimeters; Hg, mercury; mg, milligrams; dL, deciliters, WHR, waist-to-hip ratio; SD, standard deviation. The mean and standard deviation were obtained by means of a frequency analysis. Normal values: BMI, 20–25 kg/m^2^; blood pressure, 120/80 mm/Hg; cholesterol, <200 mg/dL; LDL, <130 mg/dL; HDL, >50 mg/dL in women, >40 mg/dL in men; triglycerides, <150 mg/dL; glucose, <100 mg/dL; creatinine, 0.7–1.3 mg/dL. * Statistical significance vs. non metabolic syndrome subjects. Significance of *p* was taken at a value < 0.05.

**Table 3:** Frequency of SNPs associated with obesity in the Northwest Mexican adult study population.

Gene	SNP	Risk Allele	MAF (%)
*TMEM18*	rs6548238	C	84.4
*GPX5*	rs445870	G	40.5
*PCSK1*	rs6235	G	31.4
*ZPR1*	rs964184	G	24.7
*ZBTB16*	rs7106340	T	24.7
*PPARG1*	rs3856806	T	5.7

Abbreviations: MAF: minor allele frequency.

**Table 4:** Association of SNPs with anthropometric characteristics.

Characteristics	Gene	SNP	Risk Allele	Effect (SE)	*p*-Value
BMI	*PCSK1*	rs6235	G	1.047 (0.367)	0.004 *
	*ZBTB16*	rs7106340	T	0.116 (0.547)	0.831
	*ZPR1*	rs964184	G	−0.107 (0.491)	0.107
	*GPX5*	rs445870	G	0.195 (0.333)	0.558
	*PPARG1*	rs3856806	T	0.228 (0.677)	0.736
	*TMEM18*	rs6548238	C	0.325 (0.501)	0.517
Weight	*PCSK1*	rs6235	G	2.186 (1.007)	0.030 *
	*ZBTB16*	rs7106340	T	1.213 (1.496)	0.418
	*ZPR1*	rs964184	G	−2.098 (1.343)	0.119
	*GPX5*	rs445870	G	0.236 (0.884)	0.789
	*PPARG1*	rs3856806	T	−0.174 (1.734)	0.92
	*TMEM18*	rs6548238	C	0.712 (1.321)	0.59
Diastolic pressure	*PCSK1*	rs6235	G	−0.317 (0.626)	0.613
	*ZBTB16*	rs7106340	T	1.680 (0.923)	0.069
	*ZPR1*	rs964184	G	0.967 (0.832)	0.246
	*GPX5*	rs445870	G	−0.315 (0.539)	0.559
	*PPARG1*	rs3856806	T	1.632 (1.111)	0.142
	*TMEM18*	rs6548238	C	0.876 (0.815)	0.283
Systolic pressure	*PCSK1*	rs6235	G	1.032 (1.360)	0.448
	*ZBTB16*	rs7106340	T	1.961 (2.012)	0.33
	*ZPR1*	rs964184	G	−1.387 (1.811)	0.444
	*GPX5*	rs445870	G	−0.086 (0.853)	0.92
	*PPARG1*	rs3856806	T	0.340 (1.811)	0.851
	*TMEM18*	rs6548238	C	3.218 (1.288)	0.013 *
Waist circumference	*PCSK1*	rs6235	G	2.275 (0.948)	0.017 *
	*ZBTB16*	rs7106340	T	0.928 (1.410)	0.511
	*ZPR1*	rs964184	G	−2.065 (1.262)	0.102
	*GPX5*	rs445870	G	0.072 (0.818)	0.93
	*PPARG1*	rs3856806	T	0.780 (1.686)	0.644
	*TMEM18*	rs6548238	C	1.691 (1.220)	0.166
WHR	*PCSK1*	rs6235	G	0.011 (0.005)	0.034 *
	*ZBTB16*	rs7106340	T	0.003 (0.007)	0.71
	*ZPR1*	rs964184	G	−0.010 (0.006)	0.13
	*GPX5*	rs445870	G	−0.001 (0.005)	0.834
	*PPARG1*	rs3856806	T	0.007 (0.009)	0.448
	*TMEM18*	rs6548238	C	0.012 (0.007)	0.068

Abbreviations: BMI: body mass index; SNP: single-nucleotide polymorphism; SE: standard error; WHR: waist-to-hip ratio. A linear regression was performed with an additive model; statistical analysis was adjusted for gender and age; *p* was significant at a value of <0.05. * Statistical significance.

**Table 5:** Association of SNPs with biochemical characteristics.

Biochemical Traits	Gene	SNP	Risk Allele	Effect (SE)	*p*-Value
Total cholesterol	*PCSK1*	rs6235	G	−2.311 (2.740)	0.399
	*ZBTB16*	rs7106340	T	−0.348 (3.999)	0.931
	*ZPR1*	rs964184	G	0.759 (3.608)	0.833
	*GPX5*	rs445870	G	−0.695 (3.122)	0.824
	*PPARG1*	rs3856806	T	14.310 (6.779)	0.035 *
	*TMEM18*	rs6548238	C	−1.832 (4.720)	0.698
LDL	*PCSK1*	rs6235	G	−1.355 (2.256)	0.549
	*ZBTB16*	rs7106340	T	0.483 (3.289)	0.883
	*ZPR1*	rs964184	G	2.397 (2.961)	0.419
	*GPX5*	rs445870	G	1.868 (2.086)	0.371
	*PPARG1*	rs3856806	T	3.249 (4.366)	0.457
	*TMEM18*	rs6548238	C	−1.573 (3.137)	0.616
HDL	*PCSK1*	rs6235	G	−1.386 (0.925)	0.135
	*ZBTB16*	rs7106340	T	0.470 (1.353)	0.729
	*ZPR1*	rs964184	G	−0.049(1.221)	0.968
	*GPX5*	rs445870	G	−1.655 (0.788)	0.036 *
	*PPARG1*	rs3856806	T	−0.739 (1.618)	0.648
	*TMEM18*	rs6548238	C	−0.882 (1.194)	0.46
Glucose	*PCSK1*	rs6235	G	−1.069 (0.755)	0.158
	*ZBTB16*	rs7106340	T	1.045 (1.103)	0.344
	*ZPR1*	rs964184	G	0.981 (0.993)	0.324
	*GPX5*	rs445870	G	−2.342 (3.095)	0.449
	*PPARG1*	rs3856806	T	−13.172 (6.835)	0.054
	*TMEM18*	rs6548238	C	3.441 (4.667)	0.461
Triglycerides	*PCSK1*	rs6235	G	−1.039 (4.171)	0.803
	*ZBTB16*	rs7106340	T	7.224 (6.077)	0.235
	*ZPR1*	rs964184	G	−10.644 (5.476)	0.053
	*GPX5*	rs445870	G	2.811 (4.929)	0.569
	*PPARG1*	rs3856806	T	4.759 (10.654)	0.655
	*TMEM18*	rs6548238	C	19.377 (7.436)	0.009 *

Abbreviations: SNP: single-nucleotide polymorphism; SE: standard error. A linear regression was performed with an additive model; statistical analysis was adjusted for gender, age, and BMI; *p* was significant at a value of <0.05. * Statistical significance.

**Table 6:** Individual association of SNPs and obesity.

Gene	SNP	Genotype	Normal Weight *n* = 247 (%)	Obesity *n* = 276 (%)	Odd Ratio (95%, CI)	*p*-Value
*PCSK1*	*rs6235*	CC	130 (52.7)	105 (38.0)	1.54 (1.22–1.92)	1.0 × 10^−04^ *
		GC	99 (40.0)	125 (45.2)		
		GG	18 (7.2)	46 (16.6)		
		G allele frequency %	27.3	39.3		
*ZBTB16*	*rs7106340*	GG	166 (67.2)	195 (70.6)	1.81 (0.86–1.63)	0.312
		GT	73 (29.6)	74 (26.8)		
		TT	8 (3.2)	7 (2.5)		
		T allele frequency %	18	15.9		
*ZPR1*	*rs964184*	CC	146 (59.1)	147 (53.5)	0.81 (0.607–1.09)	0.171
		GC	88 (35.6)	108 (39.3)		
		GG	13 (5.3)	21 (7.6)		
		G allele frequency %	23	27.1		
*GPX5*	*rs445870*	AA	91 (36.8)	94 (34.0)	1.22 (0.94–1.57)	0.128
		AG	125 (50.6)	125 (45.2)		
		GG	31 (12.5)	57 (20.6)		
		G allele frequency %	37.8	43.2		
*PPARG1^#^*	*rs3856806*	CC	214 (86.6)	127 (83.0)	1.78 (1.01–3.10)	0.044 *
		CT	30 (12.1)	23 (15.0)		
		TT	1 (0.4)	3 (2.0)		
		T allele frequency %	6.4	9.4		
*TMEM18*	*rs6548238*	AA	6 (2.5)	3 (1.1)	1.17 (0.80–1.70)	0.406
		GA	61 (24.6)	64 (23.1)		
		GG	180 (73.8)	209 (76.8)		
		G allele frequency %	85.2	87.3		

Abbreviations: CI: confidence index. The *p*-values and odds ratios were calculated using a logistic regression; *p*-values were adjusted for gender, age, and locality, using an additive model; the Hardy–Weinberg equilibrium was used to calculate the allele frequency; *p* was significant at a value of <0.05. * Statistical significance. *PPARG1*^#^: normal weight *n* = 245, obesity *n* = 153.

**Table 7:** Individual association of SNPs and metabolic syndrome.

Gene	SNP	Genotype	Nonmetabolic Syndrome *n* = 147 (%)	Metabolic Syndrome *n* = 376 (%)	Odd Ratio (95%, CI)	*p*-Value
*PCSK1*	*rs6235*	CC	236 (62.7)	73 (49.6)	1.50 (1.15–1.97)	3.0 × 10^−3^ *
		GC	60 (15.9)	42 (28.5)		
		GG	80 (21.2)	32 (21.7)		
		G allele frequency %	29.2	36.0		
*ZBTB16*	*rs7106340*	GG	264 (70.2)	106 (72.1)	1.32 (0.90–1.93)	0.15
		GT	96 (25.5)	34 (23.1)		
		TT	16 (4.2)	7 (4.7)		
		T allele frequency %	17.0	16.3		
*ZPR1*	*rs964184*	CC	220 (58.5)	76 (51.7)	0.78 (0.56–1.07)	0.12
		GC	141 (37.5)	54 (36.7)		
		GG	15 (3.9)	17 (11.5)		
		G allele frequency %	37.5	29.9		
*GPX5*	*rs445870*	AA	130 (34.9)	55 (37.4)	0.93 (0.75–1.15)	0.51
		AG	194 (51.9)	56 (38.0)		
		GG	52 (13.8)	36 (24.4)		
		G allele frequency %	39.2	43.5		
*PPARG1^#^*	*rs3856806*	CC	256 (87.0)	85 (81.7)	0.85 (0.55–1.32)	0.46
		CT	36 (12.2)	17 (16.3)		
		TT	2 (0.6)	2 (1.9)		
		T allele frequency %	6.8	8.6		
*TMEM18*	*rs6548238*	AA	7 (1.8)	3 (2.0)	0.87 (0.63–1.21)	0.42
		GA	38 (10.1)	15 (10.2)		
		GG	331 (88.0)	129 (87.5)		
		G allele frequency %	93.0	92.8		

Abbreviations: SNP, single-nucleotide polymorphism; CI, confidence index. The *p*-values and odds ratios were calculated using a logistic regression; *p*-values were adjusted for gender, age, and locality using an additive model; patients were classified with metabolic syndrome according to the criteria of Adult Treatment Panel III (ATP III) and American Heart Association/National Heart, Lung, and Blood Institute Scientific Statement; people with metabolic syndrome were compared with those without it; *p*-values were tested under an additive model. * Statistical significance. *PPARG1*^#^; nonmetabolic syndrome *n* = 209, metabolic syndrome *n* = 104.

## Data Availability

Data is contained within the article or Supplementary Materials.

## Supplementary Materials

The following supporting information can be downloaded at: https://www.mdpi.com/article/10.3390/genes14091775/s1, Table S1: Probe TaqMan sequence used to identify the six SNP in this study.
