# Supplementary material for: Association of PCSK1 and PPARG1 Allelic Variants with Obesity and Metabolic Syndrome in Mexican Adults
Source: Genes (Basel). 2023 Sep 8;14(9):1775. doi: 10.3390/genes14091775 (PMC10531047; doi:10.3390/genes14091775)
Supplement: Supplementary file 1 [file genes-14-01775-s001.zip › genes-2581351-supplementary.pdf]

Supplementary Table S1. Probe TaqMan sequence used to identify the six SNP in this study.

Gene	SNP	Probe sequence
PCSK1	rs6235	CAGTTTCTCTCATCGCATCATCCTG[G/T]TCTTCCTCAGAGGCTTCTGAGAACA
ZBTB16	rs7106340	CAGTTTCTCTCATCGCATCATCCTG[G/T]TCTTCCTCAGAGGCTTCTGAGAACA
ZPR1	rs964184	TCACCATCTGATGTACTGTTTTCCT[C/G]ATCTGTTTATTGTCATTTTTCCCCA
GPX5	rs445870	TGTGGAAGTTTTAAGGATGGATAAA[A/G]TCAGAGAGTTATCAGAGAAACATAA
PPARG1	rs3856806	ACCTCAGACAGATTGTCACGGAACA[C/T]GTGCAGCTACTGCAGGTGATCAAGA
TMEM18	rs6548238	AAGTCCACAGCTGGGAGCACAGGGA[C/T]TCGGGTGACTTATGCTGGGGCCTAT

Abbreviations: SNP: Single nucleotide polymorphisms. TaqMan probes were obtained from https://www.thermofisher.com.
